# Public informed training: Developing a curriculum for trialists using health systems data

**DOI:** 10.23889/ijpds.v9i5.2718

**Published:** 2024-09-18

**Authors:** Fiona Lugg-Widger, Rob Trubey, Mike Robling, Marion Mafham, Matthew Sydes, Victoria Shepherd, Rebecca Cannings-John, Kerenza Hood, Nagheen Ahmed, Sadman Islam, Sarah Rawlinson

**Affiliations:** 1 Centre for Trials Research, Cardiff University; 2 DECIPHER, Cardiff University; 3 Oxford Population Health, University of Oxford; 4 MRC Clinical Trials Unit at UCL, Institute of Clinical Trials and Methodology, UCL; 5 Centre for Trials Research, Cardiff University

## Introduction

Researchers are increasingly using health systems data to support clinical trials. A recent Delphi study identified a number of remaining challenges across the trial lifecycle and highlighted opportunities to address them through the development of training resources.

## Objective and Approach

We present a programme of work to identify, facilitate and optimise emerging practices for using health systems data in trials, informed by subject matter experts and steered by public input, to begin addressing the ongoing challenges for trial teams.

## Results

Directly addressing a key identified priority for building public trust, we co-developed a series of freely-available online training videos on how public involvement and engagement can impact across the trial lifecycle plus practical tips for such activities. This curriculum was informed by public members who then featured in the training videos. We broadened the training focus to align with trials community needs, whilst retaining the public co-production element. A diverse and purposive group of applicants were selected to contribute to the ongoing work of the HDR UK Transforming Data for Trials workstream. Our presentation reviews how models of involvement have informed our approach including reflections from our public members on what has worked well and what can be improved. We will reflect on the intersection of public involvement in trials and public involvement in healthcare/administrative systems data research.

## Conclusions & Implications

Co-development with a diverse public group ensures all current and future training resources developed reflect a diversity of views, increasing the quality and credibility of materials.

